# Supplementary material for: Synergistic and antagonistic effects of immunomodulatory drugs on the action of antifungals against Candida glabrata and Saccharomyces cerevisiae
Source: PeerJ. 2018 Jun 13;6:e4999. doi: 10.7717/peerj.4999 (PMC6004109; doi:10.7717/peerj.4999)
Supplement: Supplemental Information 4 [file peerj-06-4999-s004.docx]

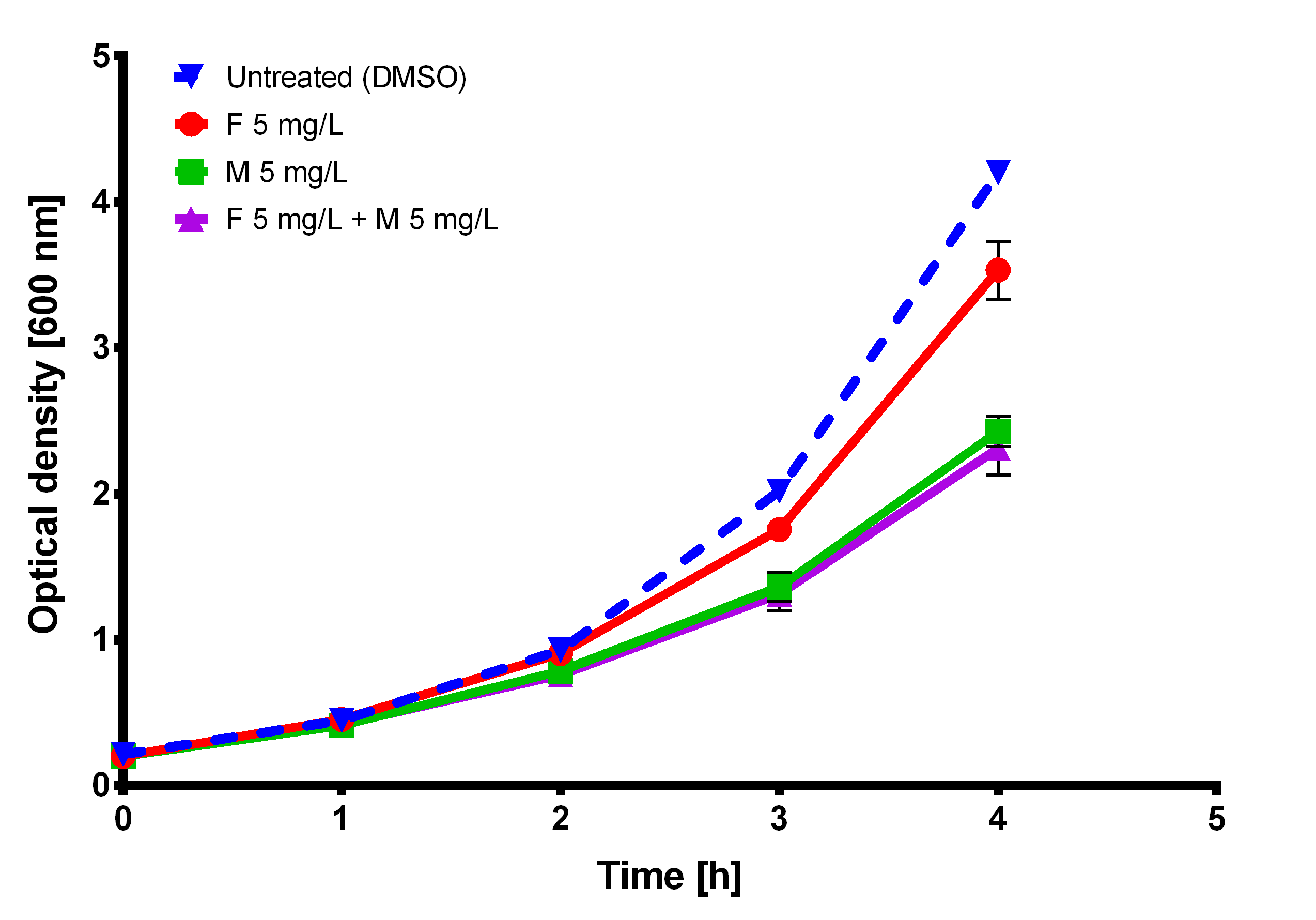


**Concentrations of fluconazole and mycophenolic acid that cause differences in *C. glabrata* ATCC 2001 growth.**

The cells were prepared as an overnight culture in liquid YPD at 37 °C. In the morning, they were diluted in fresh YPD to optical density at 600 nm (OD_600_) 0.05 and left to grow at 37 °C back OD_600_ 0.1. At that point the drugs were added and we started to monitor the changes in OD_600_. The OD_600_ is shown, as well as the standard deviation. The figure shows one representative experiment with three independent biological replicates. We were looking for the first time point, where the growth difference in response to either of the drugs or the combination of both was visible, since we have a mechanism with induced tolerance by cells responding to drugs.

F – fluconazole, M – mycophenolic acid

The first time point was determined at 2 h and the next at 4 h. This was backed-up with previous studies, where they observed *PDR1* and *CDR1* expression at similar time points with similar preparation of cells, for example 2.5 h for *PDR1* and *CDR1* in (Vermitsky et al., 2006) or 2.2 and 4.4 h in (Kuo et al., 2010) where they studied a response to drugs.

Kuo D., Tan K., Zinman G., Ravasi T., Bar-Joseph Z., Ideker T. 2010. Evolutionary divergence in the fungal response to fluconazole revealed by soft clustering. *Genome Biology* 11:R77. DOI: 10.1186/gb-2010-11-7-r77.

Vermitsky J-P., Earhart KD., Smith WL., Homayouni R., Edlind TD., Rogers PD. 2006. Pdr1 regulates multidrug resistance in Candida glabrata: gene disruption and genome-wide expression studies. *Molecular microbiology* 61:704–22. DOI: 10.1111/j.1365-2958.2006.05235.x.
